# Non-contact Sleep/Wake Monitoring Using Impulse-Radio Ultrawideband Radar in Neonates

**DOI:** 10.3389/fped.2021.782623

**Published:** 2021-12-21

**Authors:** Won Hyuk Lee, Seung Hyun Kim, Jae Yoon Na, Young-Hyo Lim, Seok Hyun Cho, Sung Ho Cho, Hyun-Kyung Park

**Affiliations:** ^1^Department of Electronics and Computer Engineering, Hanyang University, Seoul, South Korea; ^2^Department of Pediatrics, Hanyang University College of Medicine, Seoul, South Korea; ^3^Division of Cardiology, Department of Internal Medicine, Hanyang University College of Medicine, Seoul, South Korea; ^4^Department of Otorhinolaryngology, Hanyang University College of Medicine, Seoul, South Korea

**Keywords:** impulse radio ultrawideband (IR-UWB) radar, non-contact sleep/wake monitoring, non-PSG-based monitoring, NICU, neonates

## Abstract

**Background:** The gold standard for sleep monitoring, polysomnography (PSG), is too obtrusive and limited for practical use with tiny infants or in neonatal intensive care unit (NICU) settings. The ability of impulse-radio ultrawideband (IR-UWB) radar, a non-contact sensing technology, to assess vital signs and fine movement asymmetry in neonates was recently demonstrated. The purpose of this study was to investigate the possibility of quantitatively distinguishing and measuring sleep/wake states in neonates using IR-UWB radar and to compare its accuracy with behavioral observation-based sleep/wake analyses using video recordings.

**Methods:** One preterm and three term neonates in the NICU were enrolled, and voluntary movements and vital signs were measured by radar at ages ranging from 2 to 27 days. Data from a video camcorder, amplitude-integrated electroencephalography (aEEG), and actigraphy were simultaneously recorded for reference. Radar signals were processed using a sleep/wake decision algorithm integrated with breathing signals and movement features.

**Results:** The average recording time for the analysis was 13.0 (7.0–20.5) h across neonates. Compared with video analyses, the sleep/wake decision algorithm for neonates correctly classified 72.2% of sleep epochs and 80.6% of wake epochs and achieved a final Cohen's kappa coefficient of 0.49 (0.41–0.59) and an overall accuracy of 75.2%.

**Conclusions:** IR-UWB radar can provide considerable accuracy regarding sleep/wake decisions in neonates, and although current performance is not yet sufficient, this study demonstrated the feasibility of its possible use in the NICU for the first time. This unobtrusive, non-contact radar technology is a promising method for monitoring sleep/wake states with vital signs in neonates.

## Introduction

Sleep is essential to brain development and maturation in infants. Premature neonates can spend up to 90% of their time sleeping, and full-term neonates sleep ~70% of the time. Sleep progression continues as preterm neonates age (1–5). In the neonatal intensive care unit (NICU) environment, clinical interventions and alarm sounds frequently disrupt preterm neonates' sleep periods. Unstable sleep/wake patterns characterized by fragmented sleep cycling and short episodes of non-rapid eye movement (NREM) sleep increase the risk of late neurological problems (6–9).

The gold standard for sleep monitoring is expert-scored polysomnography (PSG). While highly accurate in the detection of sleep/wake states and cycles in infants, it can be an extremely challenging procedure for neonates and is limited by the need to attach adhesive electrodes or patches to the body by numerous cables (10–12). Since the classification of sleep stages cannot be judged solely based on electroencephalography (EEG) in neonates and infants, behavioral correlations of sleep and physical activity are required to distinguish each sleep state (12–14). Several criteria or guidelines for scoring sleep/wake states in neonates based on EEG patterns, respiration regularity, and other behavioral observations have been introduced, but there no widely accepted or used standards because most methods are time-consuming, laborious, obtrusive, and costly (11, 15–19). These limitations have created demand for simplified scoring of infants' sleep based solely on behavioral observations and physiological parameters such as body movements and respiration rather than EEG (10, 20–23). Changes in the regularity of the breathing rate, which are reflected in both the frequency and amplitude of breathing signals, are relatively steady during NREM sleep (12). Novel techniques of radar, capacitive electrocardiography, ballistocardiography, and laser Doppler vibrometry (12, 24) have been suggested for monitoring body movements and respiration.

Impulse-radio ultrawideband (IR-UWB) radar is a high-precision electromagnetic sensor that recognizes the motion of an object at a distance. The advantages of IR-UWB in medical applications for neonates, such as non-contact and wireless use, low exposure risk for the human body, and daily convenient use in and out of the hospital (25–28). Recently, we demonstrated that cardiorespiratory monitoring is feasible in the NICU, even though neonates' heartbeat signals are smaller and more rapid than those of adults (29–31). Because the gold standard, PSG, is not always available in limited-resource settings and requires implementation by medical professionals, sleep/wake analysis was first attempted as part of a more extended application using radar in the NICU. Radar is suitable for sleep studies that require observation for a long time and can simultaneously measure neonatal movements and vital signs such as heart rate and breathing rate while analyzing sleep/wake states (12, 32).

Our practical challenge was to integrate movements and breathing signals using IR-UWB radar with a sleep/wake decision algorithm for use with neonates in the NICU. A secondary aim was to automatically identify sleep/wake states and to evaluate their agreement with behavioral observation-based sleep/wake analyses using video recordings.

## Materials and Methods

### Subjects

We prospectively enrolled four neonates (1 preterm and 3 full-term neonates) who were admitted to the NICU of Hanyang University Hospital from March 2021 to July 2021. Neonates who had congenital anomalies or unstable medical conditions, such as fever (>38.0°C), dyspnea, high-grade intraventricular hemorrhage, and seizures, were excluded because frequent contact by medical staff would disrupt continuous data collection. The study protocol adhered to the Declaration of Helsinki and was approved by the Institutional Review Board of Hanyang University Hospital, Seoul, Korea (No. 2017-09-046-002). Written consent was given by the neonates' parents.

### Experimental Setup

**Figure 1:**
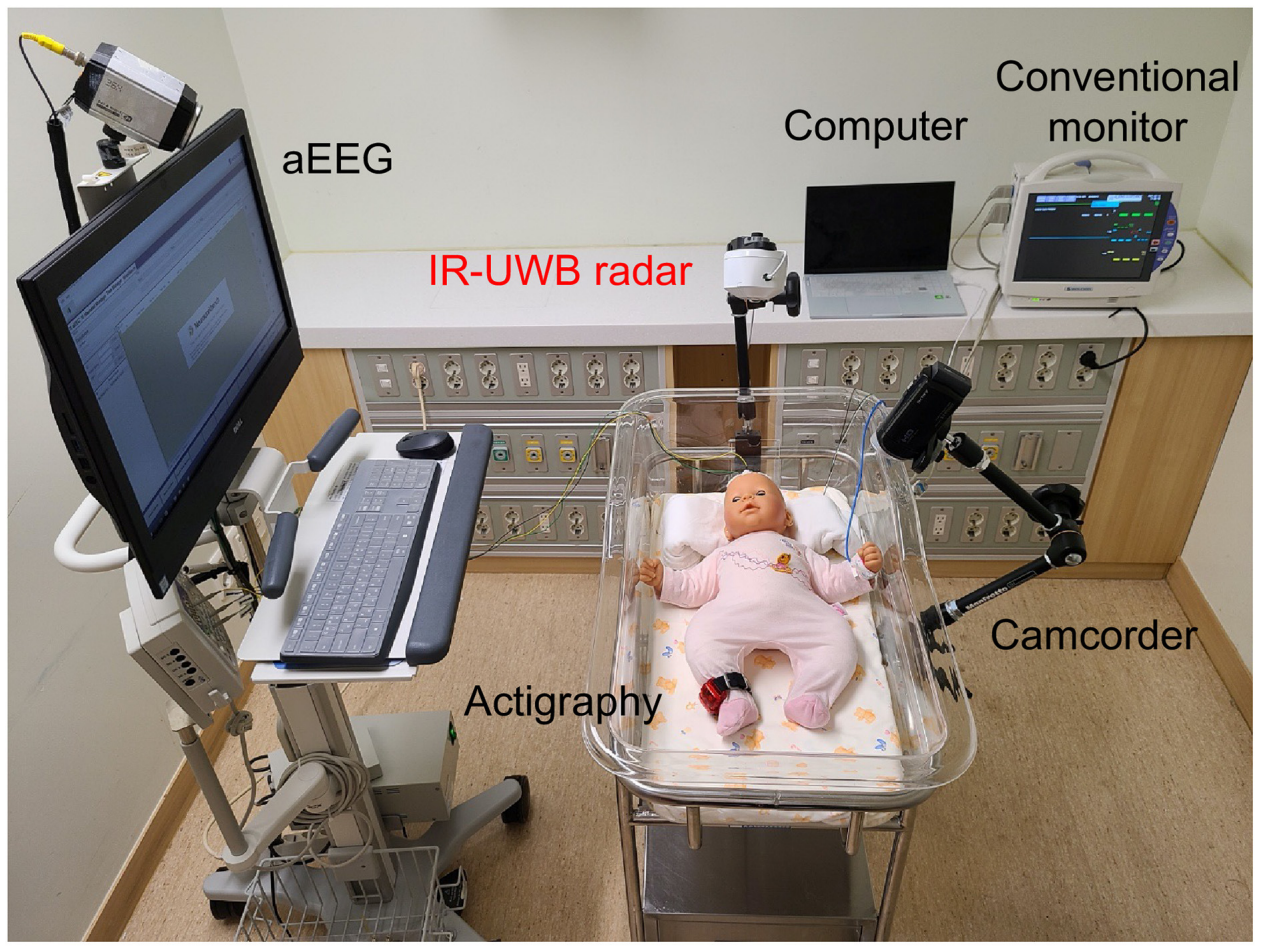
Experimental setup for simultaneous and continuous IR-UWB radar, actigraphy, and video recording. The radar sensor was covered with a cap (width × depth × height, 5.8 × 3.4 × 1.8 cm; weight, 150 g; actual sensor chip: 2.2 × 1.2 × 0.6 cm and 18 g), placed on an arm attached to the cradle and pointed at the chest of the neonate at a perpendicular angle. The sampling rate of the radar measurement was 40 Hz. A camcorder (HDR-CX450, Sony Corporation, China) was placed on the arm attached to the cradle, and the experiment was recorded. Actigraphy sensors (wGT3X-BT, Actigraphy, Florida, US) were attached to the neonate's right ankle. aEEG (EEG-1250, Nihon Kohden, Japan) was used, and 2-channel electrodes were placed on the neonate (F3-Fz-F4 and C3-Cz-C4). The neonate remained clothed during the measurements. A conventional monitor (BSM-6501k, Nihon Kohden, Tokyo, Japan) was used to measure the breathing rate and heart rate of the neonates.

The experiment was conducted in an isolated room in the NICU suitable for sleep observation with minimal light and noise. Data from the IR-UWB radar, a conventional vital sign monitor, amplitude-integrated electroencephalography (aEEG), actigraphy, and video camcorder were simultaneously recorded, as shown in Figure 1. Each neonate had minimal clothing and blankets, thus allowing relatively free movement, and was placed in a supine position in the infant cradle or incubator. The radar chip was covered with a cylindrical plastic column and placed at the end of a flexible arm on a tripod, which was held at a distance of ~40 cm from the neonate's body in a vertical upward direction. The data obtained from the radar were processed and stored on a laptop computer placed in the vicinity.

A patient monitor (BSM-6501K, Nihon Kohden, Tokyo, Japan) was used as a reference for comparison with the breathing signal from the radar as previously described in detail (29, 30), and an actigraphy sensor (wGT3X-BT, Actigraph, Florida, USA) on the neonate's right leg was used as a reference for measuring movements. The amount of motion measured with actigraphy was extracted through ActiLife (Actigraph, Florida, US) software, and the vector magnitude value was extracted at a 1-s sampling rate. Sleep/wake cycling, derived from repetitive changes between continuous and discontinuous EEG activity (33, 34), was validated using cyclical EEG patterns from aEEG (EEG-1250, Nihon Kohden, Tokyo, Japan) with 2 EEG channels (F3-Fz-F4 and C3-Cz-C4). The video camcorder (HDR-CX450, Sony Corporation, China) for behavioral observation was installed next to the radar, facing the neonate's body.

### Radar Data Collection

A commercially available IR-UWB radar sensor, XK301 (Xandar Kardian, Delaware, USA), transmitted and collected impulse signals to and from the neonate's body. In this experiment, while complying with the FCC mask range (US Federal Communications Commission Mask Regulation), the center frequency was 8.748 GHz, and the −10 dB bandwidth of 1.5 GHz was used (35–38). The radial output of the radar sensor was 68.85 μ*W* (−11.62 dBm), and the receiver was sampled at 23.328 GS/s through an analog-to-digital converter embedded in the sensor. Due to the high sampling rate, the sampled signal had a range resolution of ~6.4 mm. The radar sensor was connected to the laptop computer, and the data were processed in MATLAB (2020a, MathWorks, Natick, MA, USA) using a signal processing algorithm. The operating system of the laptop computer was Windows 10, and the number of frames per second (FPS) received from the radar sensor was 40.

### Sleep/Wake Decision

#### Video-Based Scoring

Behavioral data (body movements, eye closure, and facial grimaces) from the video, which was recorded at 1,080 p at 30 FPS, were analyzed for each 15-s epoch, and sleep/wake states were assessed and scored by a trained pediatrician based on the American Academy of Sleep Medicine (AASM) Manual for the Scoring of Sleep and Associated Events version 2.6 (19) and previous studies (10, 16, 18, 39, 40). All events, including caretaking by medical staff in the NICU, were recorded. Wake was defined as behavioral activity with the eyes open, crying or actively feeding, which are clear indicators that score as a wake state in video review. All epochs that were not scored as a wake state were categorized as a sleep state.

#### Radar-Based Scoring

##### Data Pre-processing

**Figure 2:**
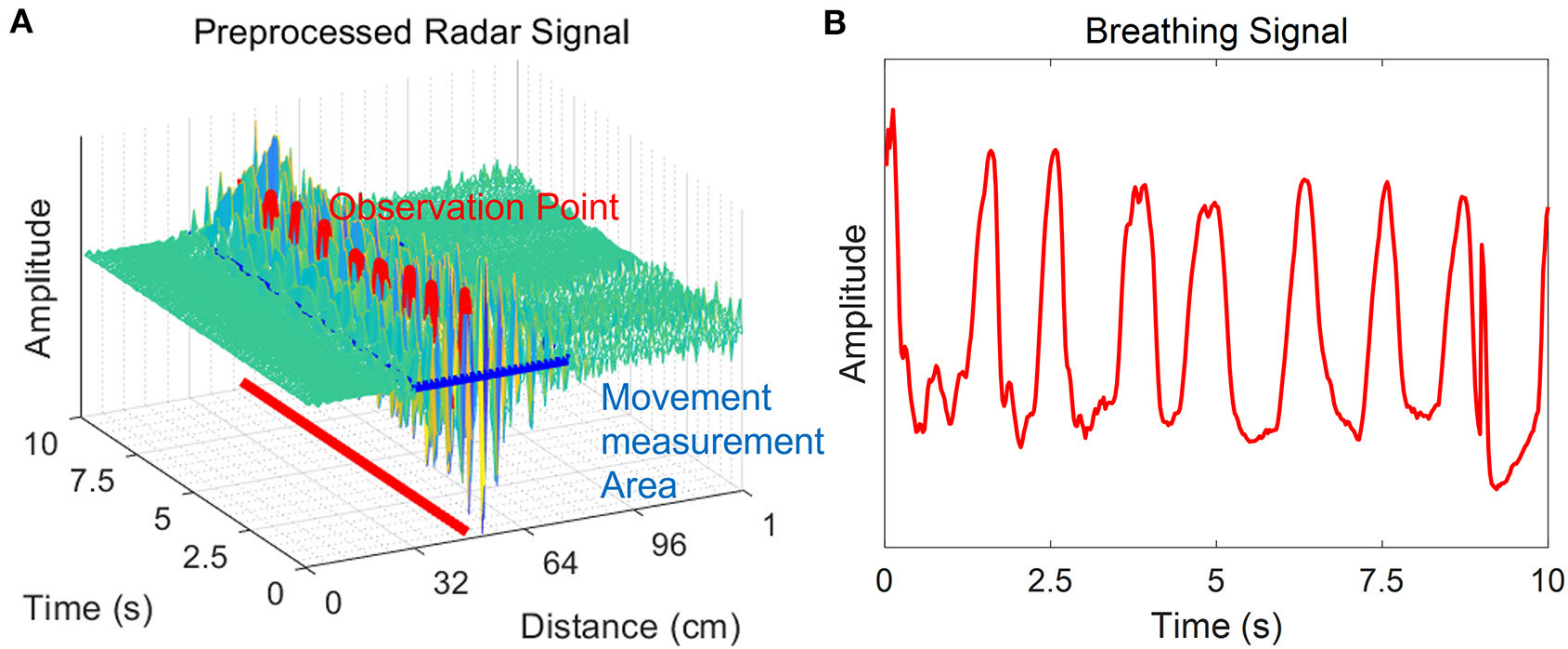
Pre-processing of the radar signal. The radar signal after bandpass filtering and application of the background subtraction algorithm is shown. The neonate is in a rest state, and there is no movement except for chest wall motion during breathing. **(A)** The x-axis is the distance from the radar to the point where the neonate's movement was measured, and the y-axis is time. The z-axis corresponds to the amplitude of the signal. The amplitude change is large in the vicinity of the observation point where the neonate was observed. The movement was quantified using pre-processed radar signals at the time of detection of infant movement. **(B)** Breathing signals were extracted from this same observation time.

Pre-processing was required to extract parameters for determining sleep/wake states of neonates from raw radar signals (Figure 2). In the received signal, many noise and clutter components were mixed with the frequency band range transmitted from the transmitter, all of which were received by the receiver. The components corresponding to noise from the received signals were removed through bandpass filtering. In addition, the received clutter components due to static objects that were present in the experimental environment were removed using a background subtraction algorithm, which is suitable for observing neonates' movements. This algorithm has been used in many previous studies to remove the signals of non-moving objects and extract only the signals of moving objects (41, 42).

##### Sleep/Wake Decision Algorithm

**Figure 3:**
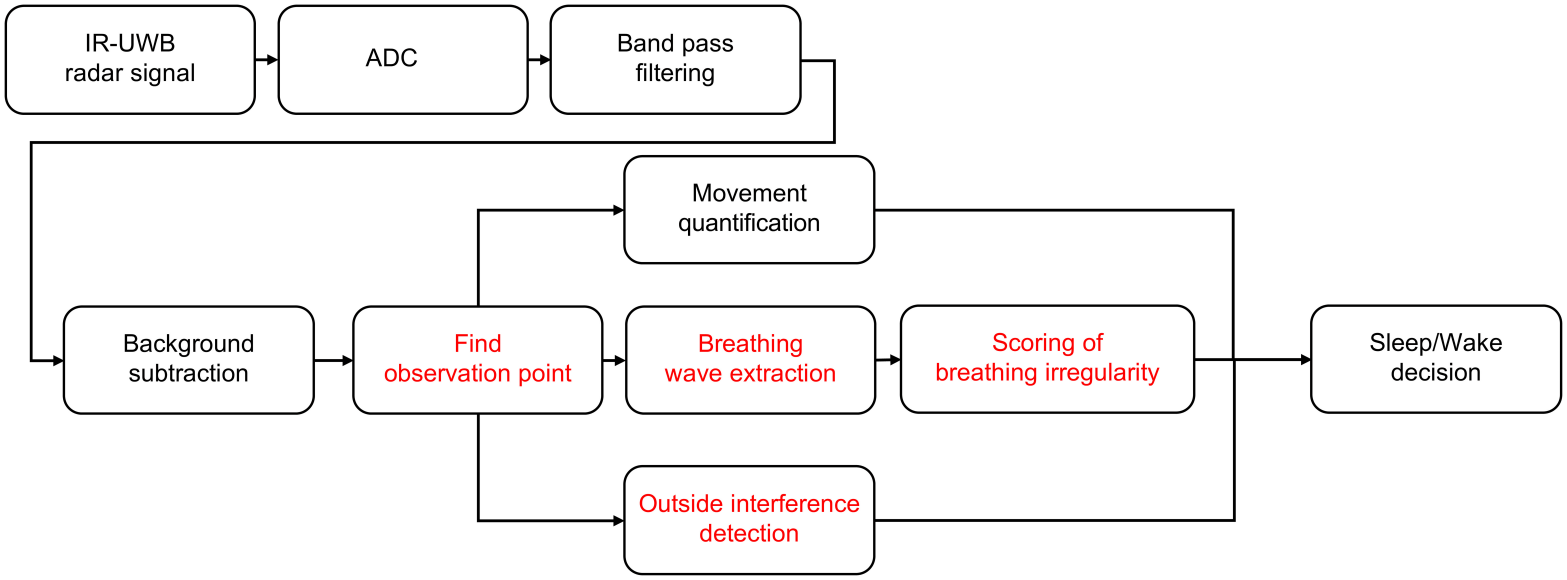
Sleep/wake decision algorithm. A sleep/wake decision algorithm designed for neonates. The noise and clutter components included in the received radar signals were removed through bandpass filters and background subtraction, and finally, neonates' sleep/wake states were determined through quantified movements, breathing wave signal quality and handling detection.

Three crucial parameters affecting neonatal sleep/wake decisions were extracted from the pre-processed radar signal and integrated into the decision algorithm: (1) quantified spontaneous movement, (2) variability in breathing rates, and (3) medical care-related motion artifacts (Figure 3). The received radar signal contained all the information, including the newborn and surrounding environmental factors, from 0 to the maximum observable distance. When a neonate is alone in an isolated room, the factors that affect the change in the radar signal are not the surrounding environmental factors but are instead the neonate's body movement or chest displacement due to breathing. Therefore, among the signals that are continuously received over time, the point with the largest change and the largest variance corresponds to the neonate's location. The corresponding point in the radar signal can identify the neonate and is most suitable for extracting the movement and biosignals of the neonate. As a result, only the non-static target signal was obtained through the background subtraction algorithm as previously described in detail (29–31). By using the background subtraction algorithm, even if the experimental environment is changed by removing the signals generated in the experimental environment, the signal used for sleep/wake decisions will show the same result if the participant does not change.

**Figure 4:**
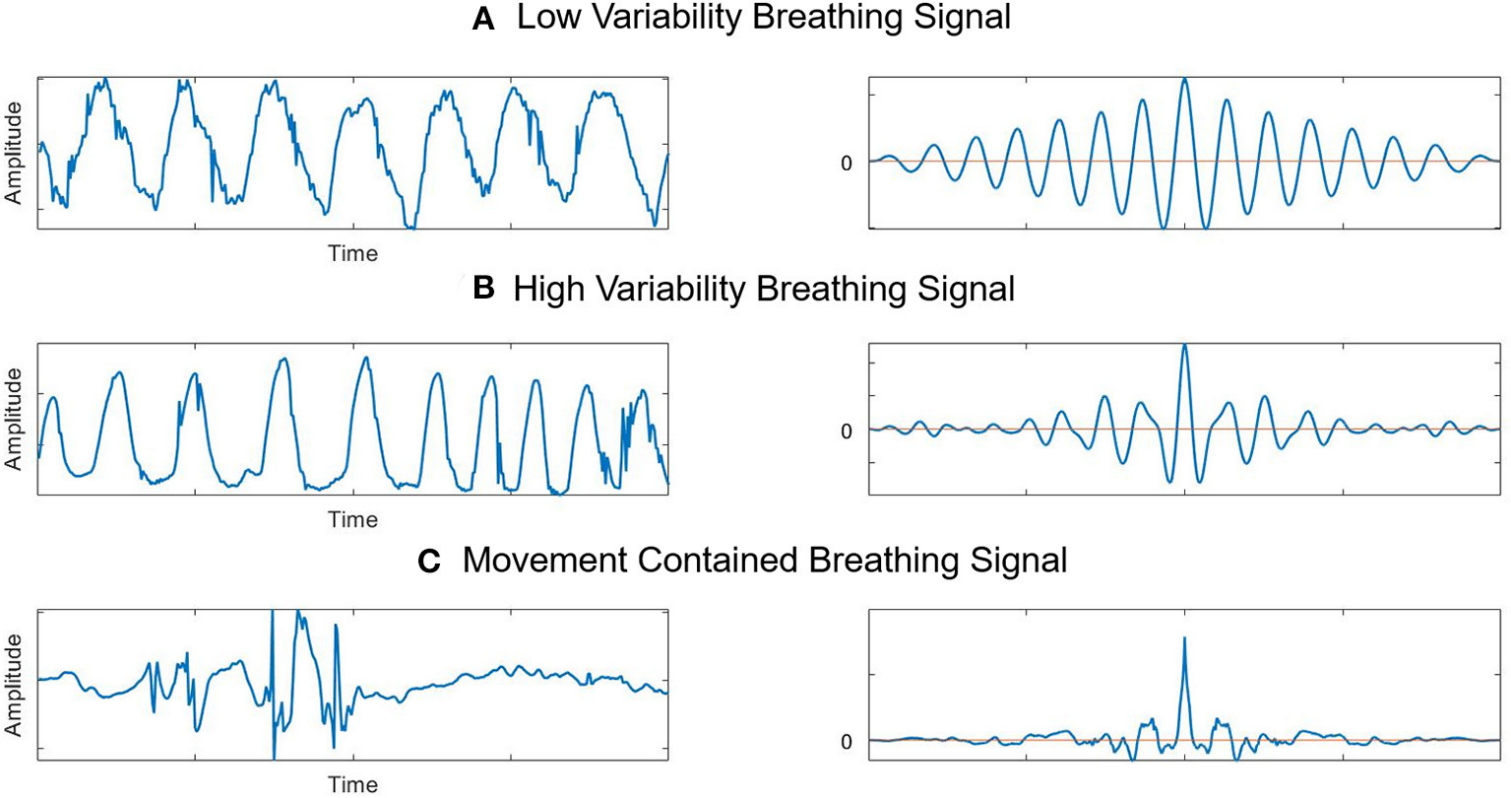
Breathing feature extraction and autocorrelations. **(A)** Stable breathing signals extracted from radar and their autocorrelation. **(B)** Breathing signals with variability and their autocorrelation. The breathing rate increased during this period, and the autocorrelation results show that zero-crossings were more frequent here than in the stable state. **(C)** Breathing signals with movements and their autocorrelation. Signals are disrupted by movement. The autocorrelation results show that there are many instances of zero-crossing.

1) *Movement measurement*: *Y*_*i*_[*k*] is the signal to which the background subtraction algorithm is applied to remove the clutter signal from the received signal. Quantified motion *Q*[*i*] is calculated by adding all the components corresponding to the difference between *Y*_*i*_[*k*] and *Y*_*i*−1_[*k*], which are pre-processed raw signals. *i* is the index corresponding to time, and *k* is the index corresponding to the distance from the radar. In the calculation process, not all components of the *Y*_*i*_[*k*] signal are used to quantify the movement; only values near the point where the neonate's movement was well-observed are used. *P* is the distance index most suitable for observing the newborn's biosignals from the received radar signal, and *offset* is a value used to observe the newborn's movement only. The greater the amount of quantified movement, the greater the probability that the newborn is awake.
  (1) $$\begin{array}{r} Q\left[i\right]=\overset{P+offset}{\underset{k=P-offset}{\sum }}g_{i}\left[k\right],g_{i}\left[k\right]={|Y}_{i}\left[k\right]-Y_{i-1}\left[k\right]| \end{array}$$
2) *Breathing features*: If the breathing rate is stable and there are no significant changes, the neonate is more likely to be sleeping. Breathing features and rates were obtained by the continuous breathing signal from the observation point in the pre-processed signal. The breathing signal-based autocorrelation feature calculation was applied to evaluate variability in the time series of breathing rates (35). In the autocorrelation of the breathing signal, a smaller number of occurrences of zero-crossing and a greater width until the first zero-crossing indicate low breathing rate variability and are counted as sleep by the radar decision algorithm (Figure 4).
3) *Medical care-related interruptions*: The effect of interruptions was a major challenge to overcome, and the radar system mistakenly classified these motions as neonatal movements during a wake state. Therefore, based on the optimized distance for observing a neonate from the radar sensor in the pre-processed signal, movement signals from outside the neonate were automatically judged as artifacts produced by medical staff in the NICU. The longer the duration of these interruptions and the greater their movement, the higher the probability that the newborn is awake. After the period of medical care, the radar algorithm resumed the same calculation for distinguishing between sleep/wake states.

Each parameter was scored for every 15-s epoch and multiplied by a weight that considered the importance of the parameter. The three parameters obtained through signal processing were synthesized, and if the score was above a certain level, the sleep/wake decision algorithm determined the state of the newborn as awake. The movement parameter was the most important weighting factor, and the disturbance signal by medical staff had the smallest weight. The radar waveform led to considerable noise in the detection of breathing signals whenever the neonate moved, preventing the efficient use of the corresponding parameter. A movement notable enough that a breathing signal was not obtained was scored as being awake.

### Statistical Analysis

Since 40 radar signals per s, i.e., 600 radar signals per epoch, were transmitted to the PC, 600 signal processing steps were required to make one sleep/wake decision. Approximately 4 min were required to execute the algorithm for 1 h of sleep/wake decisions. The data annotated in every 15-s epoch from the radar decision algorithm were compared to scoring reviewed from a reference video. A confusion matrix for each neonate was constructed, and accuracy and Cohen's kappa coefficient of agreement for sleep/wake states were extracted.

## Results

The demographics of the subjects are summarized in Table 1. Four neonates (1 preterm and 3 full-term neonates) were included. The mean birth weight and body weight at the time of the experiment were 2,482.5 (1,720–3,270) g and 2,640 (2,130–3,200) g, respectively. [Neonates can lose 4–7% of their birth weight physiologically between ~3 and 5 days of age (43), so there has been a case in which maximum weight reduced at the time of the experiment compared to birth.] All experiments occurred between 2 and 27 days after birth. The average recording time for analysis across neonates was 13.4 (8.35–20.5) h.

**Table 1:** Baseline characteristics of the subjects.

**Neonate^**a**^ number**	**Demographics at birth**				**Experimental data**					
	**Gestation (weeks)**	**Birth weight (grams)**	**SGA infant**	**Sex**	**Age (day)**	**Weight (grams)**	**Incubator**	**Respiratory support**	**Feeding^**b**^**	**Brain lesion^**c**^**
Term 1	40^+6^	2,720	AGA	Female	4	2,810	Cradle	None	Full feeding	Normal
Term 2	38^+2^	2,220	SGA	Female	10	2,420	Warmer	None	Full feeding	Normal
Term 3	37^+3^	3,270	AGA	Male	2	3,200	Cradle	None	Full feeding	Normal
Preterm 1	31^+2^	1,720	SGA	Male	27	2,130	Cradle	None	Full feeding	GMH grade 1

a *Term, baby born after 37 weeks of gestation; preterm, baby born before 37 completed weeks of gestation*.

b *Full feeding, only orally fed without intravenous nutritional support*.

c *Brain lesion identified through transcranial ultrasonography or brain magnetic resonance imaging*.

*AGA, appropriate for gestational age; SGA, small for gestational age (<10th percentile); GMH, germinal matrix hemorrhage*.

**Figure 5:**
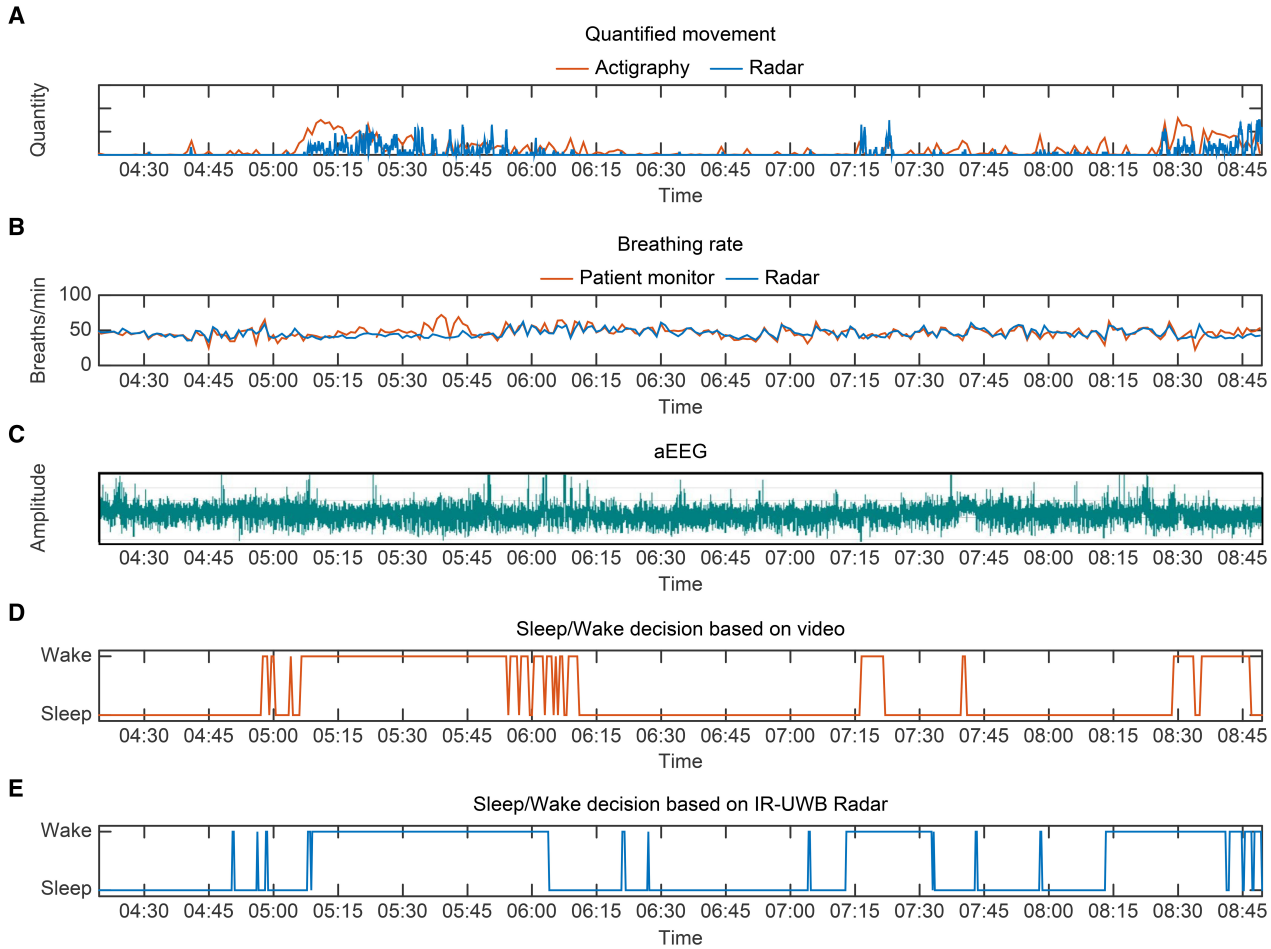
Representative sleep/wake decision diagrams from preterm 1, a 27-day-old preterm neonate weighing 2,130 g. **(A)** Quantified movement by radar and actigraphy (as a reference) over a period of 4 h 20 min. The vector magnitude measured in actigraphy and the degree of movement quantified from the IR-UWB radar are scaled for comparison. **(B)** Breathing rate measurements by the radar and conventional monitor. **(C)** aEEG from preterm 1 during the same period. Although sinusoidal variations in developed sleep/wake cycling could not be identified, continuous and discontinuous EEG backgrounds were observed, indicating immature sleep/wake cycling of preterm neonates. **(D)** Sleep/wake decision based on video review. **(E)** Sleep/wake decision using radar.

A representative case regarding the comparative flow diagram for sleep/wake decisions by four different methods is depicted in Figure 5. This neonate was born prematurely at 31^+2^ weeks' gestation weighing 1,700 g, and she was 27 days old and weighed 2,130 g at the time of the experiment. Before performing pairwise comparisons, we first investigated whether the radar was highly concordant in measuring the main parameters for sleep/wake decisions, namely, movements and breathing rates. For reference, actigraphy and conventional patient monitor were used. The vector magnitude measured in the actigraphy sensor and the degree of movement quantified from the IR-UWB radar, which is presented in arbitrary units based on the distance from the radar, were compared. The upper two tachograms show that the measurements of quantified movements and breathing rates using the two different methods were similar, and the middle tachogram shows neonatal aEEG during the experiment (Figures 5A–C). Although sinusoidal variations in developed sleep/wake cycling could not be identified, continuous and discontinuous EEG backgrounds were observed, indicating immature sleep/wake cycling of preterm neonates. In the two bottom panels, sleep/wake decisions by the radar algorithm are compared to the actual scoring based on the review from the video recordings (Figures 5D,E). These diagrams represent the similarities between the two methods, and as expected, minor discrepancies occurred when the neonate moved with her eyes closed (sleep state) or when she did not move with her eyes open (wake state).

A total of 12,464 data points from 15-s epochs for all neonates were analyzed for comparisons between the radar and video review. The output data, accuracy and Cohen's kappa for sleep/wake decisions are presented in Table 2. The total video recording time was 51.9 h, and the average recording time was 13.0 (7.0–20.5) h. The percentage of sleep time during the experiment was 53.3% (46.3–59.5) by radar and 64.2% (56.0–71.8) by video. Wake state agreement (0.81) was more consistent than sleep state agreement (0.72) across all neonates. Preterm 1 had the highest correlation, with a sleep state agreement of 0.79, a wake state agreement of 0.84, and an overall agreement of 0.81.

**Table 2:** Individual agreement of sleep/wake classification between IR-UWB radar and video review.

**Neonate number**	**Recording time (hours)**	**Total epoch**	**Sleep (epoch)**		**Wake (epoch)**		**Sleep time (%)**		**Total recall**	**Cohen's kappa (κ)**
			**Radar/Video**	**Recall**	**Radar/Video**	**Recall**	**Radar**	**Video**		
Term 1	8.67	2,080	827/1,306	0.63	636/774	0.82	46.4	62.8	0.70	0.41
Term 2	7	1,680	877/1,206	0.73	351/474	0.74	59.5	71.8	0.73	0.41
Term 3	15.75	3,780	1,394/2,118	0.66	1,304/1,662	0.78	46.3	56.0	0.71	0.43
Preterm 1	20.52	4,924	2,678/3,366	0.80	1,312/1,558	0.84	59.4	68.4	0.81	0.59

For all neonates, we achieved a mean Cohen's kappa of 0.49 (0.41–0.59), a moderate agreement level, and an overall accuracy of 0.75 (0.70–0.81) in an epoch-by-epoch comparison for sleep/wake decisions between the radar and video review (Table 3).

**Table 3:** Overall accuracy of sleep/wake classification between IR-UWB radar and video review.

**Cohen's kappa (κ) 0.4956**	**Video**		
	**Sleep**	**Wake**	**Recall**
**IR-UWB radar**			
Sleep	5,776	865	0.87
Wake	2,220	3,603	0.62
Precision	0.72	0.81	0.75

## Discussion

For the first time, our innovative non-contact technology, IR-UWB radar, successfully distinguished neonatal sleep/wake states based on changes in movements and breathing signals and provided a high degree of accuracy compared with behavioral observation through video recordings, one of the main methods for monitoring the sleep of neonates. Moreover, we suggest that IR-UWB radar is a feasible non-contact technique to continuously monitor neonates' sleep in the NICU. Although signal interruptions produced by medical staff and overestimation/underestimation issues from misjudgment based on neonates' movement information are major challenges to overcome, we successfully distinguished sleep/wake states using a new decision algorithm for neonates.

Sleep is important in early brain development, and poor-quality sleep in premature and term neonates has lasting effects on later cognitive functioning (15). In particular, rapid eye movement (REM) sleep in premature infants is a critical component that provides ascending stimulation to the forebrain to promote brain development when wake-related stimulation is low and topographic mapping of the vertebral cortex is occurring. Strategies to promote neonates' sleep have been implemented as developmental care in NICUs (3, 5, 44). Today, infant sleep is scored using two main methods: PSG and behavioral observations (12). PSG uses a number of measurements that require the attachment of sensors and electrodes to the infant's body to measure brain activity, eye movements, heart rate, heart rate variability, muscle activity, and respiration. The use of adhesive electrodes on preterm neonates can cause damage to their fragile skin, making them more prone to infections (45). For that reason, PSG is a very challenging procedure for neonates and cannot be performed in tiny premature neonates, even in the NICU setting. The necessity of sleep monitoring and the obtrusiveness of this commonly used method lead us to argue that unobtrusive sleep measurement methods are required for a comfortable alternative that results in minimal stress on neonates. Capacitive electrocardiogram (ECG), video camera, laser Doppler vibrometry, ballistocardiography, and actigraphy are useful (9, 12, 24, 46) but are time-consuming and not continuous, and most medical staff are not trained to interpret EEG or aEEG.

The IR-UWB radar sensor used in this study is a non-invasive, non-contact, wireless, continuous and unobtrusive diagnostic device for monitoring neonates' breathing and movement (29, 30). In addition, radars are readily available, even with limited healthcare resources, and have many advantages, including daily convenience, low cost, high data throughput, and long-term applicability for predicting neurological deficits. We have recently used IR-UWB radar to measure sleep and sleep apnea and compared it with PSG with good performance in adults (31, 47). However, studies validating radar sleep data against PSG or behavioral scoring are still extremely limited, especially in neonates. We introduced an algorithm for simultaneously measuring neonates' movement, breathing, and heart rate (29, 30, 41), and the radar data suggested the possibility of distinguishing between sleep/wake states in neonates since many sleep studies have focused on extracting informative features from breathing signals and movements (48). The advantages of these radars suggest that neonates' sleep/wake states can be evaluated not only in hospitals but also in homes. In addition, due to its relatively small size, radar can be installed without interference from peripheral devices, especially in the NICU.

Our results showed that IR-UWB radar can accurately detect sleep/wake states in a non-contact manner. Although frequent medical care-related interruptions affected our ability to obtain high-quality signals of movement and breathing, the accuracy was sufficiently high. Accuracy and Cohen's kappa with regard to sleep/wake decisions were highest for a preterm neonate (preterm 1). Based on the overall recordings, these results can be attributed to the longer experiment duration for a preterm neonate, which accordingly included more night time. In the NICU environment at night, ambient stimuli such as noise are decreased, and the ratio of stable sleep/wake cycles increases. In fact, there was no significant difference among the neonates in the percentage of sleep time in video review (Table 2), but preterm 1 had the lowest number of wake events. The majority of sleep for a neonate is REM sleep until ~3 months post-term. The ratio of REM sleep appeared to be higher in the other neonates than in preterm 1. Since there are body movements when neonates are in the REM sleep state, a higher ratio of REM sleep resulted in discrepancies when the radar made sleep state decisions.

This study has several limitations. First, the study was conducted in the NICU and not under laboratory conditions conducive to a sleep study. In the NICU, there is uncontrolled noise in the hospital environment, such as monitoring devices, which acts as a disruptive factor for neonates' sleep. This creates unwanted movements of the neonates and results in a bias that lowers the accuracy of the measurements. Second, IR-UWB radar was used only to collect movement and respiratory information to determine the neonates' sleep/wake states. In some situations, it is difficult to determine the sleep/wake state of neonates using only information obtained from IR-UWB radar. The radar will judge situations of drowsiness in which the neonate's eyes are open but movements are of low magnitude as a sleep state because of the lack of movement; however, the neonate is actually in a wake state. In a situation where the neonate is sleeping but shows a startle or muscle twitch, the radar would identify the neonate as is in a wake state.

Future work could focus on the classification of each sleep state of REM and NREM sleep and the interpretation of additional sleep parameters such as total sleep time, sleep efficiency, and sleep apnea through improved radar techniques. Further optimization in the NICU and modality fusion at the sensor level are needed to enhance the performance of neonatal sleep/wake monitoring.

## Conclusions

IR-UWB radar can provide considerable accuracy in the sleep/wake analysis of neonates, and this study is the first to provide data supporting the feasibility of this non-contact and continuous method in the NICU. These results are promising for the future use of the radar technique by clinicians as a screening diagnostic tool during early life to recognize neonates' sleep problems, provide individualized support to maintain sleep quality, and detect sleep disorders that are associated with motor activity.

## Data Availability Statement

The original contributions presented in the study are included in the article/supplementary material, further inquiries can be directed to the corresponding authors.

## Ethics Statement

The studies involving human participants were reviewed and approved by Institutional Review Board of Hanyang University Hospital, Seoul, Korea. Written informed consent to participate in this study was provided by the participants' legal guardian/next of kin.

## Author Contributions

WHL, SHK, JYN, SHoC, and HKP: had full access to all of the data in the study and take responsibility for the integrity of the data and the accuracy of the data analysis. WHL, SHK, and HKP: study concept and design. WHL and SHoC: acquisition, analysis, or interpretation of data. SHK and JYN: drafting of the manuscript. WHL and SHK: statistical analysis. SHoC and HKP: obtained funding, park administrative, technical, or material support. YHL, SHyC, SHoC, and HKP: study supervision. All authors critical revision of the manuscript for important intellectual content.

## Funding

This research was supported by the Bio and Medical Technology Development Program (Next Generation Biotechnology) through the National Research Foundation of Korea (NRF) funded by the Ministry of Science, ICT and Future Planning (NRF-2017M3A9E2064735), and the research fund of Hanyang University MEB (Global Center for Developmental Disorders, HY-201900000003070).

## Conflict of Interest

The authors declare that the research was conducted in the absence of any commercial or financial relationships that could be construed as a potential conflict of interest.

## Publisher's Note

All claims expressed in this article are solely those of the authors and do not necessarily represent those of their affiliated organizations, or those of the publisher, the editors and the reviewers. Any product that may be evaluated in this article, or claim that may be made by its manufacturer, is not guaranteed or endorsed by the publisher.
